# Frailty phenotype state transitions among older adults with a history of cancer and diabetes

**DOI:** 10.1186/s12877-025-06309-6

**Published:** 2025-08-14

**Authors:** Anna G. Kuzma, Hannah N. Lee, Heidi D. Klepin, Emilie D. Duchesneau

**Affiliations:** 1 Department of Epidemiology and Prevention, Division of Public Health Sciences, Wake Forest School of Medicine, 475 Vine St, Winston-Salem, NC 27101 USA; 2 Department of Internal Medicine, Section on Hematology and Oncology, Wake Forest School of Medicine, Winston-Salem, NC USA

**Keywords:** Multistate model, Longitudinal, Nationally representative, Frailty, Cancer, Diabetes

## Abstract

**Background:**

Frailty is a multi-faceted, aging-related syndrome characterized by reduced physiologic reserve and increased vulnerability to adverse health outcomes. Frailty is dynamic and can improve or worsen over time. Cancer and diabetes are prevalent comorbid conditions in older adults that are independently associated with frailty. Little is known about how the co-occurrence of cancer and diabetes affects frailty in older adults. We investigated how co-occurring cancer and diabetes influence long-term frailty state transitions in older adults.

**Methods:**

We conducted a longitudinal study using Rounds 1–9 of the National Health and Aging Trends Study (2011–2019), a nationally-representative prospective cohort of Medicare beneficiaries (65 + years). We described nine-year transitions across frailty phenotype states (robust, prefrail, frail) and death using a multi-state model, stratifying by cancer and diabetes history in Round 1: neither condition (*N* = 4,216), cancer only (*N* = 972), diabetes only (*N* = 1,434), and both conditions (*N* = 331). We compared the nine-year incidence of frailty across subgroups using Aalen-Johansen estimators, adjusting for age, race, and gender.

**Results:**

Older adults without a history of cancer or diabetes had the lowest nine-year mortality (57.5%) compared to those with cancer only (64.4%), diabetes only (65.1%), or both conditions (64.9%). The prevalence of frailty among those alive across time periods was highest in older adults with cancer and diabetes (Round 1 [R1]: 24.7%, R9: 22.3%) and those with diabetes only (R1: 24.0%, R9: 22.4%). The prevalence of frailty was lower in participants with cancer only (R1: 18.0%, R9: 16.3%) and those with neither condition (R1: 13.1%, R9: 15.1%). Among participants who were robust or prefrail at baseline, the nine-year cumulative incidence of frailty was higher among those with diabetes only (52.8%) or diabetes and cancer (54.6%) compared to those with cancer only (41.4%) or neither condition (47.3%).

**Conclusions:**

Diabetes and cancer were both associated with an increased frailty prevalence; however, only older adults with diabetes or cooccurring diabetes and cancer had an increased 9-year incidence of frailty relative to older adults without a history of either condition. Future research should explore the underlying mechanisms driving frailty state transitions among chronic disease populations and evaluate targeted interventions to mitigate frailty progression.

**Supplementary Information:**

The online version contains supplementary material available at 10.1186/s12877-025-06309-6.

## Introduction

Due to advances in cancer treatment and care delivery, the population of cancer survivors is rapidly aging [1]. Diabetes is a common comorbid condition in older cancer survivors, with approximately one-quarter having previously been diagnosed with diabetes at the time of their cancer diagnosis [2]. Comorbid diabetes has been shown to increase the risk and stage of cancers as well as the risk of cancer recurrence [3]. It is crucial to understand how comorbid cancer and diabetes affect other patient-centered outcomes in this growing and medically-complex population.

Frailty is a multi-faceted syndrome characterized by reduced physiologic reserve and increased vulnerability to adverse health outcomes [4]. Frailty is dynamic and can improve or worsen over time, although worsening is more common in older adults [5]. Frailty has been recognized as a major burden to cancer treatment, outcomes, and quality of life in older cancer survivors [6, 7]. In addition, cancer and its treatments contribute to elevated occurrence of frailty in older survivors [8]. Older adults with diabetes are also more likely to become frail, and frail older adults with diabetes are more likely to die or experience diabetes complications than their counterparts [9]. 

Although previous studies have demonstrated the individual impacts of cancer and diabetes on frailty, none have investigated how these conditions jointly influence long-term transitions in frailty state transitions [1, 10]. This gap is particularly important, as the co-occurrence of both conditions may produce synergistic effects on physiological decline. Understanding these relationships can help inform intervention development to improve long-term health, well-being, and maintenance of independence in this population [11, 12]. 

To fill this research gap, our study leverages data from a nationally representative prospective cohort of older adults to understand long-term frailty state transitions in older adults with comorbid cancer and diabetes. We compare these outcomes to older adults with a history of cancer only, a history of diabetes only, or without either condition. We hypothesized that older adults with a history of cancer, diabetes, or both conditions would have higher prevalence and incidence of frailty compared to those with neither condition and that the prevalence and incidence would be largest among those with a history of both conditions.

## Methods

### Data source

We analyzed longitudinal data from Rounds 1–9 (2011–2019) of the National Health and Aging Trends Study (NHATS). NHATS is a prospective cohort designed to investigate trends in disability, physical functioning, and aging in older adults in the United States [13, 14]. The study conducts annual in-home interviews for a nationally representative sample of over 8,000 Medicare beneficiaries aged 65 and older. While at the time of writing NHATS data were available through Round 12 (2022), we only included data through Round 9 because the performance-based measures used to identify frailty (grip strength and walking speed tests) were not captured in Round 10 (2020) due to the COVID-19 pandemic. Our study included all older adults who completed the primary NHATS data collection instrument (the Sample Person interview) in 2011. We excluded older adults with missing cancer or diabetes history in Round 1 (*N* = 8) and those with a history of skin cancer (*N* = 648). Those with a history of skin cancer were excluded due to the high prevalence of non-aggressive skin cancers that behave differently than other cancers, which could skew results for those with a cancer history. Since NHATS does not differentiate between malignant melanoma and other skin cancers in their public use files, those with malignant melanoma were also excluded.

### Frailty

Frailty was measured annually from Rounds 1–9 using the Fried frailty phenotype [4]. The Fried frailty phenotype defines frailty as a clinical, aging-related syndrome based on the presence of five clinical signs or symptoms: exhaustion, low physical activity, shrinking, slowness, and weakness. We assessed the five frailty phenotype components in NHATS using self-reported and performance-based measures based on definitions from prior research (e.g., slowness and weakness are based on grip strength and walking trials, respectively) [11]. Consistent with the original definition, older adults who met definitions for three to five frailty phenotype components were considered “frail”, those with one to two components were considered “prefrail”, and those with zero were considered “robust” [4]. 

### Cancer and diabetes history

Cancer history was determined based on responses to the following question during the initial NHATS interview: “Has a doctor ever told you that you had cancer”? Although NHATS collects additional details on the specific type of cancer, these are not available in the public use files and were not available for our analysis. Individuals with a history of diabetes during the initial NHATS interview were identified through their responses to the survey question: “Has a doctor ever told you that you had diabetes?” We stratified all our analyses using the following categories: neither condition, cancer only, diabetes only, or both cancer and diabetes.

### Other variables

Death is determined in NHATS by attempted follow up and confirmation from a proxy respondent or family member [13]. We identified several covariates in the NHATS data to account for differences in demographic and clinical characteristics by cancer and diabetes history and to account for potentially informative missing data. These variables included socioeconomic status (wealth and educational attainment), demographics (age, self-reported race and ethnicity, gender, metro/non-metro residence, and marital status), BMI, use of mobility devices, and prior history of myocardial infarction, heart disease, high blood pressure, arthritis, recent falls, osteoporosis, lung disease, stroke, depression, and anxiety [13, 15, 16]. 

### Statistical analysis

This study received expedited approval from the Wake Forest School of Medicine Institutional Review Board (#IRB00108630). All analyses were conducted using SAS Version 9.4 (SAS Institute Inc., Cary NC) and R Version 4.2.3.

#### Missing data

Fifteen percent of individuals were missing frailty information in Round 1. We accounted for missing frailty and covariate data using multiple imputation with fully conditional specification (also called multiple imputation with chained equations) [17, 18]. This missing data approach fits a series of iterative predictive models to multiply impute each variable with missing data. Our imputation models included the outcome (frailty) and stratification variables (cancer and diabetes history), demographics (age, race, gender), socioeconomic status (wealth, educational attainment), other comorbidities, history of falls, and use of mobility devices. We generated ten multiply imputed datasets using ten burn-in iterations. All statistical analyses were conducted within each multiply imputed dataset and then combined to generate a single parameter estimate using Rubin’s rule [19]. Since cancer and diabetes history had a low degree of missingness (*N* = 8), we excluded those with missing cancer and diabetes information, rather than imputing these variables.

#### Standardization

We accounted for differences in the age, race, and gender distributions across cancer and diabetes strata using standardization via standardized mortality ratio (SMR) weighting [20]. Older adults with a history of both cancer and diabetes served as the standard, as the primary goal of our study was to draw inference to those with comorbid cancer and diabetes. SMR weights were calculated separately within each multiply imputed dataset. All statistical analyses incorporated both the SMR weights and the NHATS survey sampling weights to draw inference to Medicare beneficiaries over 65 years of age in 2011.

#### Baseline prefrailty and frailty prevalence differences

We estimated the difference in the prevalence of prefrailty and frailty during Round 1 for older adults with cancer only, diabetes only, a history of both cancer and diabetes, or neither condition using an SMR- and survey-sample weighted multinomial regression model with the frailty phenotype as the dependent variables (robust was the reference group) and cancer and diabetes strata as the explanatory variable. Older adults without a history of cancer or diabetes were the reference group for comparison.

#### Multistate model

We described longitudinal frailty state transitions across the three frailty phenotype categories (robust, prefrail, and frail) and death between NHATS rounds using an interval-censored, nonparametric multistate model [21]. We implemented the multistate model using a two-step approach. First, the risk of mortality over time was estimated using an SMR- and survey-sample weighted Kaplan-Meier estimator (using the svykm function in R) to account for right censoring due to loss-to-follow-up in NHATS [22]. Then, the proportion of individuals within each frailty phenotype state among those alive in each round was non-parametrically calculated based on the observed data. The multistate models were fit separately within the four cancer and diabetes strata.

#### Cumulative incidence of prefrailty and frailty

We estimated the nine-year cumulative incidence of (1) prefrailty or frailty and (2) frailty using SMR- and survey-sample weighted Aalen-Johansen estimators that accounted for death as a competing risk and censoring due to loss-to-follow-up [23, 24]. Those who were robust in Round 1 of NHATS served as the study population in the analysis of the cumulative incidence of prefrailty or frailty, and those who were robust or prefrail in Round 1 were included in the analysis of the cumulative incidence of frailty. These study populations were selected to represent the populations at risk for prefrailty and frailty, respectively. 95% confidence intervals were estimated based on the 2.5th and 97.5th percentiles from 1,000 bootstrapped samples [25]. 

We conducted a sensitivity analysis that considered a composite outcome of frailty or death over the nine-year period. The time to the first occurrence of frailty or death was considered the event. The cumulative incidence of frailty or death, among those who were robust or prefrail at baseline, was estimated using an SMR- and survey-sample weighted Kaplan-Meier estimator, stratified by cancer and diabetes history.

## Results

### Study population

We included 6,953 older adults in the NHATS cohort: 4,216 (60.6%) had no history of cancer or diabetes, 972 (14.0%) had a history of cancer only, 1,434 (20.6%) had a history of diabetes only, and 331 (4.8%) had a history of both cancer and diabetes (Table 1). On average older adults with a history of cancer or a history of cancer and diabetes were older than those with diabetes only or neither condition. A higher proportion of older adults with a history of diabetes alone or cancer and diabetes were Black (34.8% and 34.3%, respectively) than those with a history of cancer only (19.7%) or no history of cancer or diabetes (20.7%). Older adults with a history of diabetes only and those with a history of both diabetes and cancer had lower median wealth ($50,394, $57,000) than those with a history of cancer only, or neither condition ($107,406, $94,500).

**Table 1:** Characteristics of NHATS participants stratified by a history of no cancer or diabetes, cancer only, diabetes only, and both cancer and diabetes, during the round 1 (2011) interview, after multiple imputation with fully conditional specification and accounting for survey-sample weighting

Characteristic	Overall *N* = 6,953	No Diabetes or Cancer History *N* = 4,216	Cancer History Only *N* = 972	Diabetes History Only *N* = 1,434	History of Cancer and Diabetes *N* = 331
Demographics, *n* (survey sample weighted %)					
* Place of residence*					
Community	6,569 (94.3)	3,995 (94.8)	909 (93.2)	1,355 (93.7)	310 (92.5)
Non-nursing home residential care	384 (5.7)	221 (5.2)	63 (6.8)	79 (6.3)	21 (7.5)
* Age*					
65–69	1,323 (28.6)	856 (30.6)	130 (20.9)	281 (28.1)	56 (25.5)
70–74	1,461 (25.0)	840 (24.0)	175 (22.9)	363 (28.7)	83 (28.9)
75–79	1,394 (19.2)	791 (17.8)	218 (22.9)	316 (20.7)	69 (19.6)
80–84	1,357 (14.3)	827 (14.3)	208 (16.6)	256 (12.5)	66 (14.8)
85–89	841 (8.8)	518 (8.8)	146 (11.4)	148 (7.5)	29 (6.4)
90+	577 (4.1)	384 (4.4)	95 (5.2)	70 (2.4)	28 (4.9)
Gender, n (survey sample weighted %)					
Female	4,142 (58.0)	2,585 (59.5)	550 (58.4)	838 (54.0)	169 (51.9)
Male	2,811 (42.0)	1,631 (40.5)	422 (41.6)	596 (416.0)	162 (48.1)
Self-reported racial and ethnic group, n (survey sample weighted %)					
Non-Hispanic White	4,604 (79.5)	2,954 (81.8)	722 (85.8)	739 (69.0)	188 (73.6)
Non-Hispanic Black	1,678 (9.2)	874 (7.7)	192 (7.4)	499 (14.3)	114 (14.4)
Hispanic	452 (7.4)	262 (6.9)	33 (3.6)	139 (11.7)	18 (7.0)
Non-Hispanic Other	218 (3.1)	126 (3.6)	25 (3.2)	57 (4.9)	11 (4.9)
Metro/non-metro, n (survey sample weighted %)					
Metropolitan area	5,681 (82.0)	3,408 (81.4)	807 (83.5)	1,192 (82.8)	274 (83.8)
Non-metropolitan area	1,272 (18.0)	808 (18.6)	164 (16.5)	242 (17.2)	57 (16.2)
Socioeconomic status					
Wealth, median (IQR)	$85,000 ($1,400, $309,000)	$94,500 ($2,000, $350,000)	$107,406 ($5,869, $380,000)	$50,394 ($500, $200,000)	$57,000 ($651, $258,500)
*Educational attainment*, n (survey sample weighted %)					
Less than high school	1,978 (22.8)	1,138 (21.4)	231 (19.2)	517 (30.1)	92 (22.1)
High school equivalent or higher	4,975 (77.2)	3,078 (78.6)	741 (80.8)	917 (69.9)	239 (77.9)
Medical history, n (survey sample weighted %)					
* Smoking history*					
Never smoker	3,457 (47.6)	2,151 (48.6)	438 (44.6)	727 (48.4)	141 (39.3)
Former smoker	2,945 (43.8)	1,716 (42.4)	468 (48.4)	603 (43.6)	157 (48.9)
Current smoker	552 (8.7)	349 (9.0)	66 (7.0)	103 (8.0)	33 (11.7)
Body mass index, median (IQR)	26.6 (23.5, 30.4)	25.9 (23.1, 29.4)	25.8 (22.9, 29.3)	29.0 (25.4, 33.1)	28.8 (25.4, 32.6)
Hospital stays in past year	1,616 (20.9)	810 (17.0)	289 (27.1)	403 (26.0)	114 (35.3)
* Comorbid conditions*					
Myocardial infarction	1,041 (13.6)	537 (11.5)	143 (12.2)	288 (20.0)	73 (20.8)
Heart disease	1,252 (16.8)	652 (14.1)	160 (15.6)	356 (25.3)	84 (21.8)
High blood pressure	4,662 (63.5)	2,574 (57.6)	649 (64.4)	1,166 (78.4)	273 (79.3)
Arthritis	3,851 (53.1)	2,208 (50.2)	545 (56.0)	898 (59.2)	200 (59.5)
Osteoporosis	1,415 (21.1)	848 (21.0)	224 (24.2)	282 (20.5)	61 (17.3)
Lung disease	1,036 (15.1)	576 (13.6)	173 (18.2)	223 (16.3)	63 (21.7)
Stroke	807 (10.0)	412 (8.2)	113 (9.6)	228 (14.9)	53 (15.2)

*Abbreviations*: *IQR* Interquartile range

^a^Ns reflect the number of individuals within the study sample, while percentages account for the NHATS survey sample weights

### Baseline prevalence of frailty

**Fig. 1:**
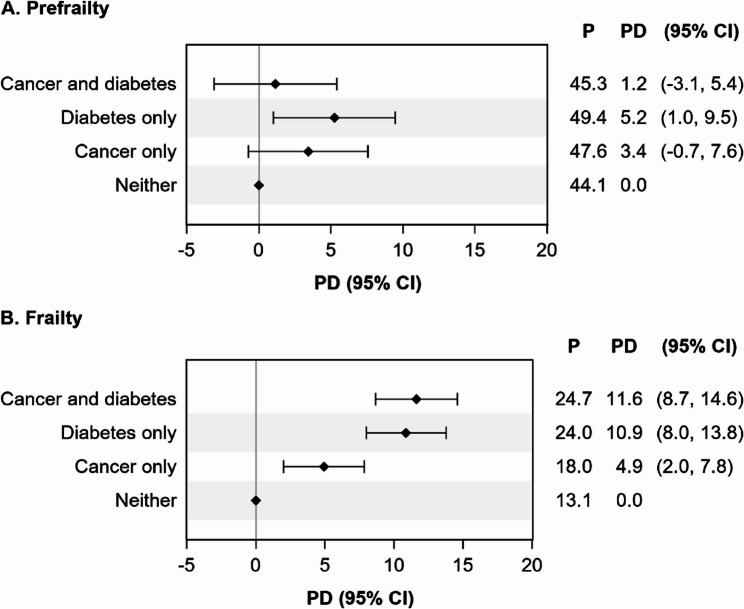
Comparison of prevalence of **A** prefrailty and **B** frailty during the baseline Round 1 NHATS interview, among older Medicare beneficiaries by cancer and diabetes history. Abbreviations: CI=confidence interval; NHATS=National Health and Aging Trends Study; P=prevalence; PD=prevalence difference. Those with “neither” condition served as the reference group when estimating prevalence differences

After accounting for differences in the age, race, and gender distribution using SMR weighting, the baseline prevalence of prefrailty during Round 1 was slightly higher in individuals with cancer, diabetes, or both conditions compared to those with neither condition (Fig. 1, Panel A), although estimates were imprecise. Alternatively, the baseline prevalence of frailty was highest among those with comorbid cancer and diabetes (24.7%), followed by those with a history of diabetes only (24.0%), those with a history of cancer only (18.0%), and those with neither condition (13.1%) (Fig. 1, Panel B).

### Frailty multi-state models

**Fig. 2:**
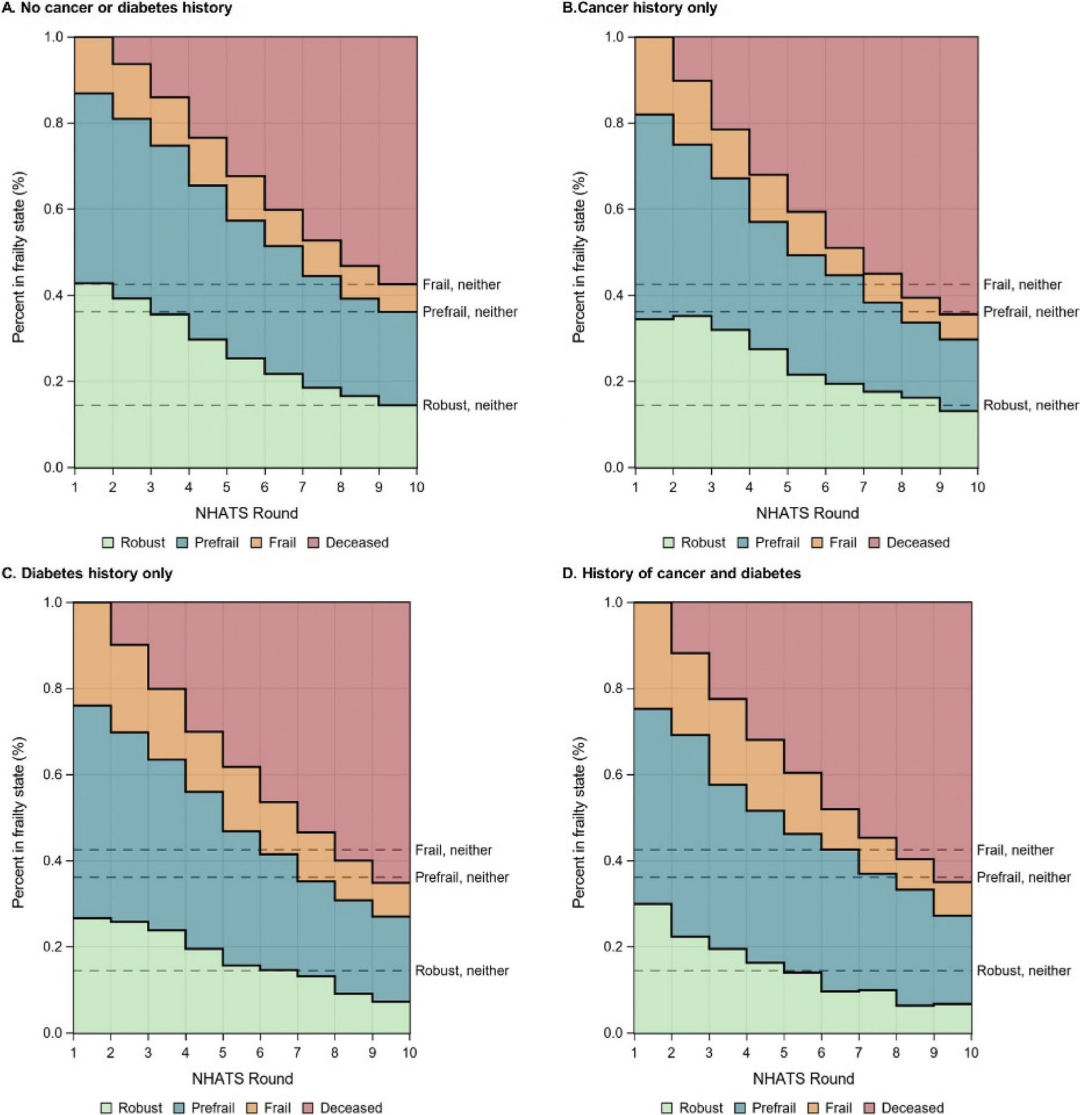
Multistate model of nine-year frailty state transitions in older Medicare beneficiaries, by cancer and diabetes history. Abbreviations: NHATS = National Health and Aging Trends Study

Individuals with neither a history of cancer nor diabetes had lower 9-year mortality (57.5%, 95% CI 53.3-61.7%) than those with cancer history only (64.4%, 95% CI 53.4-75.4%), diabetes history only (65.1%, 95% CI 57.3-72.9%), or a history of both cancer and diabetes (64.9%, 95% CI 48.1-81.8%), although confidence intervals were wide due to small sample sizes (Fig. 2, Supplemental Table 1). The proportion of individuals who were robust across the nine-year follow-up period was highest among those without a history of cancer or diabetes (Round 1: 42.8%, Round 9: 14.4%), followed by those with a history of cancer only (Round 1: 34.4%, Round 9: 13.1%). Alternatively, among those who remained alive, the proportion of individuals who were prefrail or frail was highest in the diabetes only (Round 1: 73.4%, Round 9: 27.6%) and diabetes and cancer strata (Round 1: 70.0%, Round 9: 28.3%).

### Cumulative incidence of prefrailty and frailty

**Fig. 3:**
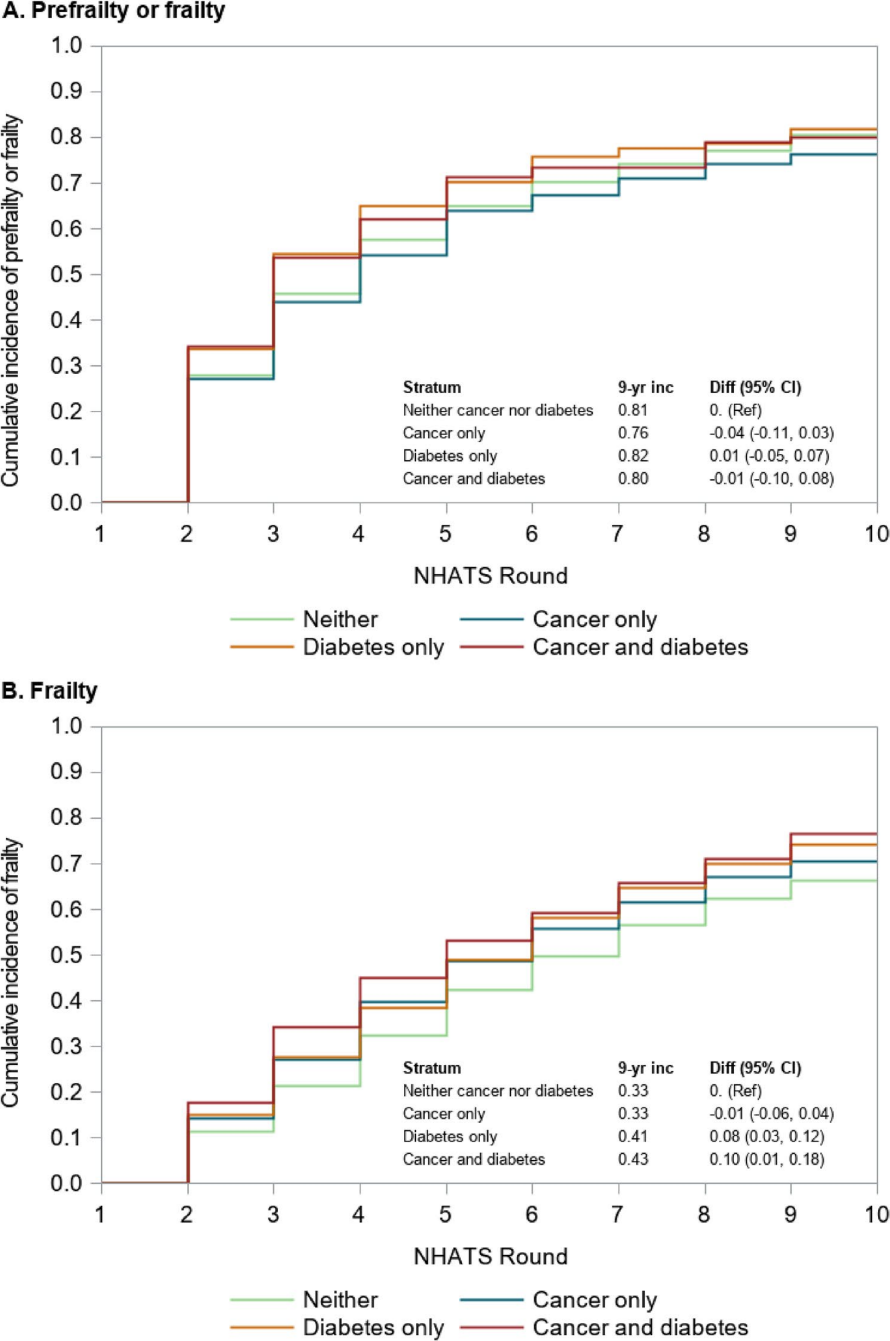
Nine-year cumulative incidence of prefrailty or frailty **A** or frailty **B** in older Medicare beneficiaries, by cancer and diabetes history. Abbreviations: CI= confidence interval; diff= difference; inc=incidence; NHATS = National Health and Aging Trends Study

Among older adults who were robust at baseline, the cumulative incidence of prefrailty or frailty was higher during the early years of follow-up among those with a history of both cancer and diabetes, as well as those with diabetes alone, compared to those with cancer only and those with neither condition (Fig. 3A). However, these differences diminished over time, and by nine years post-baseline, the cumulative incidence of prefrailty or frailty had converged across groups, with most participants becoming prefrail or frail (cumulative incidence range from 76 to 81%).

Among older adults who were robust or prefrail at baseline, the nine-year cumulative incidence of frailty was highest among those with a history of cancer and diabetes (43%) and those with a history of diabetes only (41%). In contrast, the nine-year cumulative incidence of frailty was lower among those with a history of cancer only (33%) and those without a history of cancer or diabetes (33%) (Fig. 3B). Together, these findings suggest that diabetes, particularly when co-occurring with cancer, is associated with an elevated risk of frailty progression from both robust and prefrail states; however, its impact on progression to prefrailty appears to be less pronounced.

**Fig. 4:**
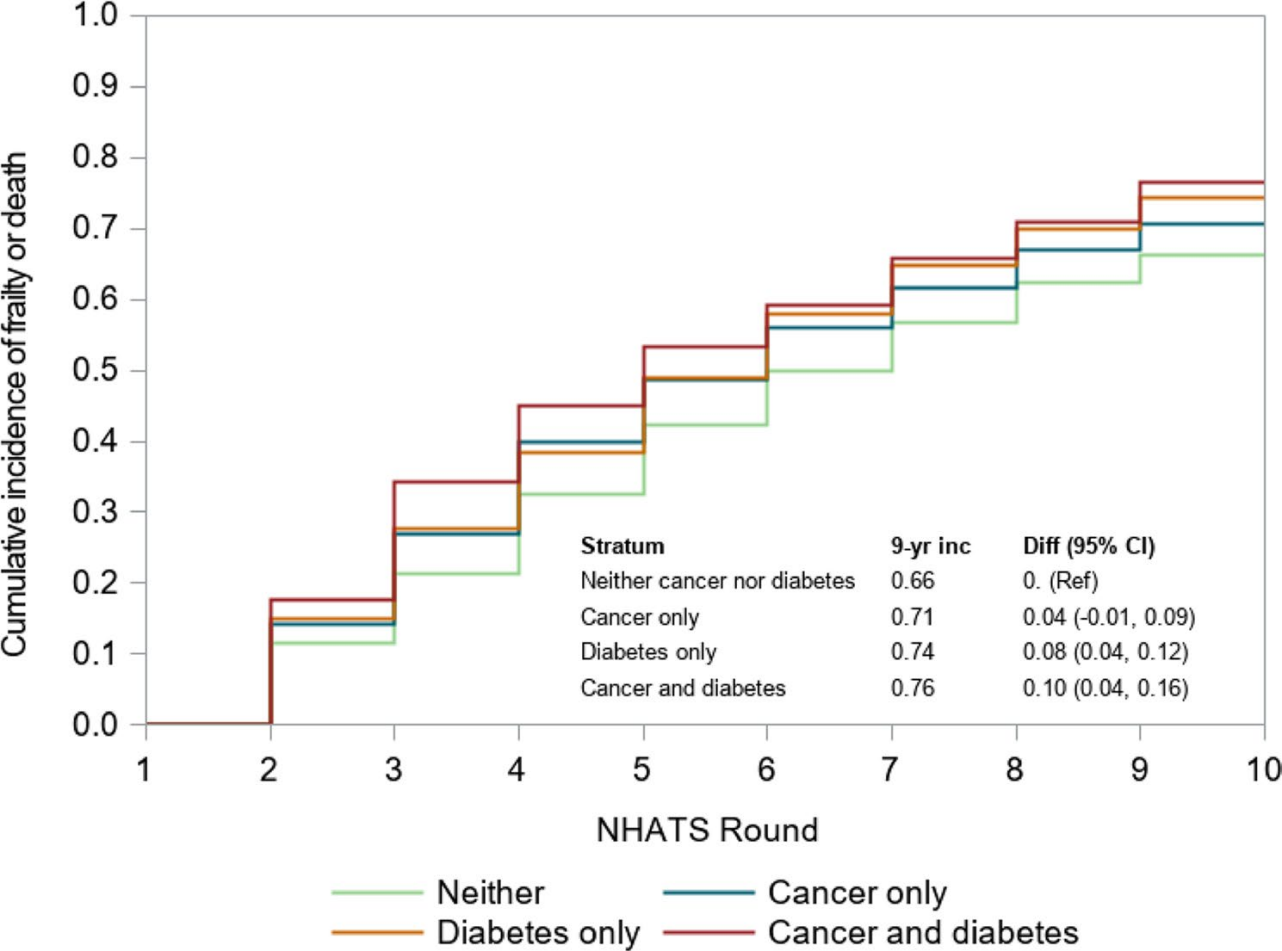
Nine-year sensitivity analysis of frailty and death in older Medicare beneficiaries in the National Health and Aging Trends Study (2011–2019), by cancer and diabetes history. Abbreviations: CI= confidence interval; diff= difference; inc=incidence; NHATS = National Health and Aging Trends Study

In our sensitivity analysis (Fig. 4), the nine-year cumulative incidence of frailty or death as a composite outcome was similar between older adults with a history of cancer (71%) and those with neither cancer nor diabetes history (66%). Alternatively, older adults with a history of diabetes only (74%) and those with a history of cancer and diabetes (76%) had the highest cumulative incidence of frailty or death.

## Discussion

Using a nationally representative sample of older Medicare beneficiaries, we found that older adults with a history of cancer, diabetes, or both had higher baseline prevalence of frailty compared to those with no history of either condition in Round 1. The prevalence of frailty was highest among older adults with comorbid cancer and diabetes and those with diabetes alone. In contrast, the baseline prevalence of prefrailty was more similar across groups, with individuals with a history of diabetes alone having modestly higher baseline prevalence of prefrailty compared to the other groups.

Older adults with a history of cancer only, diabetes only, or both conditions had similar elevated nine-year all-cause mortality compared to those without a history of either condition. However, the proportion of individuals who were robust after nine years was lower, and the proportions who were prefrail or frail were higher, in older adults with a history of diabetes only and those with a history of diabetes and cancer, compared to those with a history of cancer only. Among older adults who were robust or prefrail at baseline, those with diabetes only or both cancer and diabetes had a higher cumulative incidence of frailty than those with cancer only or neither condition. In contrast, the nine-year cumulative incidence of a combined outcome of prefrailty or frailty among those robust at baseline was similar across groups. These findings suggest that differences in longitudinal frailty transitions by diabetes and cancer history are largely driven by differences in the incidence of frailty, rather than prefrailty.

Diabetes and cancer are two prevalent chronic conditions in the aging population and understanding their impact on long term health is crucial to improving outcomes and promoting healthy aging [2, 26]. Across all analyses, diabetes appeared to be a stronger contributing factor to long-term frailty than cancer, indicated by an increased prevalence and incidence throughout the nine-year follow-up. This was contrary to our hypothesis that the comorbid cancer and diabetes subgroup would have the worst frailty outcomes. The influence of diabetes on frailty may be attributed to its systemic nature that affects multiple body systems [26]. The increased risk of frailty may arise from interactions between coexisting medical conditions, such as obesity and cardiovascular disease, along with the deterioration of muscle and nerve function, decreased cardiopulmonary reserve, loss of executive function, and a prolonged state of chronic inflammation [27, 28]. Insulin resistance related to poor glucose control is hypothesized to be a major source of muscle loss due to its role as an anabolic hormone, leading to diminished physical health [28]. Additionally, patients with diabetes exhibit a chronic state of heightened inflammation caused by increased inflammatory mediators and an imbalance of pro- and anti-inflammatory factors [27, 29]. These interconnected mechanisms may collectively influence the development and progression of frailty over time [28].

Older adults with a history of cancer alone had a higher prevalence of frailty and risk of mortality compared to older adults with neither cancer nor diabetes throughout the follow-up period. The increased prevalence of frailty may be due to detrimental effects of cancer and its treatments on frailty and physical function that persist during the survivorship period. Surprisingly, the cumulative incidence of frailty was lower in older adults with a history of cancer only than in those with a history of neither cancer nor diabetes (41% vs. 47%). Older adults who do not become frail during the acute phase surrounding cancer diagnosis and treatment may not be at increased risk of developing frailty during the survivorship phase, relative to the general population without a history of cancer. Another potential explanation for this finding may be our choice to treat death as a competing risk in the primary analysis and the higher incidence of mortality among those with a history of cancer than those without a history of cancer. Older adults who died before becoming frail (or before being observed as frail in NHATS) were removed from the risk set. We further tested this hypothesis by conducting a sensitivity analysis that considered death or frailty as a composite outcome, which found a similar cumulative incidence between the group with a history of cancer only and a history of neither condition. Alternatively, our study may be impacted by survival bias, with older cancer survivors who survive long enough to be observed in NHATS representing a healthier cohort than all cancer survivors.

A limitation of our study was an inability to identify the timing of cancer or diabetes diagnoses, cancer type and stage, diabetes type and severity, diabetic control, and use of anticancer and antidiabetic medications in the NHATS data. For example, older adults with a history of cancer may have recently experienced their cancer diagnosis during older age or may be years removed from diagnosis and treatment. This lack of timing information may partially explain why we did not observe an increased cumulative incidence of frailty for older adults with cancer compared to older adults without a history of cancer or diabetes. Furthermore, diabetes has been shown to be associated with increased risk of certain types of cancers, such as liver, pancreatic, endometrial and GI malignancies, and we may expect synergistic associations between cancer and diabetes on frailty to differ in these cancer types [30]. Despite these limitations, our research is novel in describing long-term frailty in older adults with a history of diabetes and cancer and can inform future studies. Additional research using patient registries, cohort studies, or data linkages, such as between NHATS and Medicare medical encounter and pharmacy claims, may help uncover the mechanisms behind our findings.

Our study should be interpreted considering other limitations. Due to NHATS data collection procedures, we excluded older adults residing in nursing homes, who have a higher prevalence of frailty than those living in the community or non-nursing home residential care settings. Our findings are not generalizable to this population. In addition, information on frailty and mortality are only assessed annually in NHATS, and we were unable to identify the exact timing of our outcome measures. The frailty phenotype components are measured in NHATS using both self-reported (exhaustion, low physical activity, shrinking) and objective measures (weakness, slowness). As with any self-reported measures, there is room for potential recall bias and misclassification [31]. However, the method to assess these variables in NHATS is consistent with the original methods used to define the frailty phenotype in the landmark study by Fried et al. [4] Thus, although the self-reported nature of some variables is a limitation, it reflects standard practice in frailty research and allows for comparability across studies. Finally, frailty had a high degree of missing data (15.4%), which we accounted for using multiple imputation. However, if our imputation models were incorrectly specified, missing data bias may remain.

## Conclusions

To our knowledge, our study is the first to model the intersectional associations between cancer and diabetes on frailty state transitions in older adults. Using a nationally representative sample, we found that both cancer and diabetes have an impact on mortality and initial frailty state in older adults, but our results highlight that diabetes seems to have a much larger effect on transitions through frailty states. This is likely attributable to the systemic and chronic nature of diabetes in comparison with the often acute and localized nature of some cancers. The population of older adults and the prevalence of diabetes and cancer continue to rise, and the intersection of these conditions is common. Clinicians treating older adults with diabetes and cancer across specialties should consider the interplays between cancer, diabetes, and frailty when managing patients to ensure healthy aging and maintenance of independence.

## Supplementary Information

Supplementary Material 1.

## Data Availability

The datasets generated and/or analyzed during the current study are available in the National Health and Aging Trends Study repository, https://nhats.org/.

## Abbreviation

NHATS: National health and aging trends study
