# Supplementary material for: Frailty phenotype state transitions among older adults with a history of cancer and diabetes
Source: BMC Geriatr. 2025 Aug 14;25:624. doi: 10.1186/s12877-025-06309-6 (PMC12351896; doi:10.1186/s12877-025-06309-6)
Supplement: Supplementary file 1 — Supplementary Material 1. [file 12877_2025_6309_MOESM1_ESM.docx]

**Supplemental Table 1: Proportion of individuals robust, prefrail, frail, and deceased over time from multistate model of nine-year frailty state transitions in older Medicare beneficiaries, by history of cancer and diabetes**

| **Strata^1^** | **Robust** | **Prefrail** | **Frail** | **Deceased** |
| --- | --- | --- | --- | --- |
| No cancer or diabetes history |  |  |  |  |
| Round 1 | 42.8% | 44.1% | 13.1% | 0.0% |
| Round 2 | 39.3% | 41.7% | 12.7% | 6.3% |
| Round 3 | 35.6% | 39.2% | 11.2% | 14.0% |
| Round 4 | 29.7% | 35.7% | 11.1% | 23.4% |
| Round 5 | 25.3% | 32.0% | 10.3% | 32.3% |
| Round 6 | 21.7% | 29.7% | 8.4% | 40.2% |
| Round 7 | 18.5% | 26.0% | 8.3% | 47.2% |
| Round 8 | 16.6% | 22.6% | 7.6% | 53.2% |
| Round 9 | 14.4% | 21.7% | 6.4% | 57.5% |
| Cancer history only |  |  |  |  |
| Round 1 | 34.4% | 47.6% | 18.0% | 0.0% |
| Round 2 | 35.2% | 39.8% | 14.8% | 10.2% |
| Round 3 | 31.9% | 35.2% | 11.4% | 21.5% |
| Round 4 | 27.5% | 29.5% | 10.9% | 32.0% |
| Round 5 | 21.6% | 27.7% | 10.1% | 40.6% |
| Round 6 | 19.4% | 25.2% | 6.4% | 49.0% |
| Round 7 | 17.6% | 20.7% | 6.7% | 55.0% |
| Round 8 | 16.2% | 17.4% | 5.8% | 60.6% |
| Round 9 | 13.1% | 16.6% | 5.8% | 64.4% |
| Diabetes history only |  |  |  |  |
| Round 1 | 26.6% | 49.4% | 24.0% | 0.0% |
| Round 2 | 25.8% | 44.0% | 20.3% | 9.9% |
| Round 3 | 23.9% | 39.6% | 16.4% | 20.1% |
| Round 4 | 19.5% | 36.5% | 14.0% | 30.0% |
| Round 5 | 15.7% | 31.2% | 14.9% | 38.2% |
| Round 6 | 14.6% | 27.0% | 12.1% | 46.4% |
| Round 7 | 13.1% | 22.1% | 11.3% | 53.4% |
| Round 8 | 9.1% | 21.7% | 9.2% | 60.0% |
| Round 9 | 7.2% | 19.8% | 7.8% | 65.1% |
| History of cancer and diabetes |  |  |  |  |
| Round 1 | 30.0% | 45.3% | 24.7% | 0.0% |
| Round 2 | 22.4% | 46.8% | 19.0% | 11.8% |
| Round 3 | 19.5% | 38.2% | 19.9% | 22.4% |
| Round 4 | 16.3% | 35.3% | 16.5% | 31.9% |
| Round 5 | 14.0% | 32.2% | 14.2% | 39.6% |
| Round 6 | 9.6% | 32.9% | 9.4% | 48.0% |
| Round 7 | 10.0% | 27.0% | 8.4% | 54.7% |
| Round 8 | 6.4% | 26.9% | 7.1% | 59.6% |
| Round 9 | 6.7% | 20.5% | 7.8% | 64.9% |
| Abbreviations: CI=confidence interval; PD=prevalence difference. | | |  |  |
| ^1^ In order to account for differences in demographic factors across strata, we standardized the age, gender, and race/ethnicity distributions in each stratum to reflect the distribution among older adults with a history of cancer and diabetes. | | | | |
|  |  |  |  |  |
|  |  |  |  |  |
